# Contextual Factors Influencing U.S. College Students' Decisions to Drink Responsibly

**DOI:** 10.3109/10826084.2012.690811

**Published:** 2012-06-04

**Authors:** Adam E. Barry, Patricia Goodson

**Affiliations:** 1 Department of Health Education & Behavior, University of Florida, Gainesville, Florida, USA; 2 Department of Health & Kinesiology, Texas A&M University, College Station, UAS

**Keywords:** alcohol, responsible drinking, contextual factors, college students, mixed-methods, harm reduction, problem drinkers

## Abstract

This mixed-methods study qualitatively (n = 13—convenience) explored contextual factors influencing decisions to drink responsibly, and quantitatively (n = 729—random) assessed the prevalence of these factors and whether they varied as a function of sex and binge-drinking status. Data were collected in 2007 among Texas college students. The constant comparison model was used to analyze the qualitative data, while one-way ANOVAs and logistic regression were employed to assess the quantitative data. Effect sizes are reported for all significant ANOVA interactions. Psychometric properties are supplied for all quantitative scales. Implication and limitations are noted, and future research directions discussed.

## RÉSUMÉ

**Les Facteurs d'après le contexte Influençant les Décisions d'Etudiants de collège américaines de Boire Responsable**

Ceci les mélangeé-méthodes étudient qualitativement (n = 13 - la convenance) a exploré des facteurs d'après le contexte qui influencent des décisions boire responsablement, et quantitativement (n = 729 - fait au hasard) a évalué la prédominance de ces facteurs et s'ils ont varié comme une fonction de sexe et comme le statut frénésie-boiant. Les données ont été recueillies dans 2007 parmi Texas étudiants de collège. Le modèle constant de comparaison a été utilisé pour analyser les données qualitatives, pendant qu'ANOVAs unilatéral et la régression logistique ont été employés pour évaluer les données quantitatives. Les tailles d'effet sont rapportées pour toutes les interactions d'ANOVA significatives. Les propriétés de Psychometric sont fournies pour toutes les échelles quantitatives. L’implication et les limitations sont notées, et les directions de recherche futures ont discuté.

**Mots clés**: L'alcool, Boire Responsable, les Facteurs d'après le contexte, Etudiants de collège, Mélangé-Méthodes

## RESUMEN

**Los Factores contextuales que Influyen EEUU Estudiantes Colegiales' las Decisiones de Beber Responsable**

Esto mezcló-métodos estudian cualitativamente (n = 13 - conveniencia) exploró los factores contextuales que influyen las decisiones de beber responsablemente, y cuantitativamente (n = 729 - aleatorio) valoró la frecuencia de estos factores y si variaron como una función de estatus de sexo y jarana-bebiendo. Los datos fueron reunidos en 2007 entre Tejas estudiantes colegiales. El modelo constante de la comparación fue utilizado para analizar los datos cualitativos, mientras de un solo sentido ANOVAs y retroceso logístico fueron empleados para valorar los datos cuantitativos. Los tamaño del efecto son informados para todas las interacciones signifcativas de ANOVA. Las propiedades psicométricas son suministradas para todas las escalas cuantitativas. La implicación y las limitaciones son notadas, y futuras direcciones de investigación discutieron.

**Palabras clave**: El alcohol, Beber Responsable, los Factores Contextuales, Estudiantes Colegiales, los Mezclado-Métodos

## INTRODUCTION

Even though 4 out of 10 U.S. college students engage in heavy episodic alcohol consumption (Johnston, O'Malley, Bachman, & Schulenburg, 2008; O'Malley & Johnston, 2002; Wechsler, Dowdall, Maenner, Gledhill-Hoyt, & Lee, 1998; Wechsler, Lee, Kuo, & Lee, 2000; Wechsler & Nelson, 2001; Wechsler et al., 2002), only a small subset consistently continues these heavy drinking patterns after college and into adulthood (Weingardt et al., 1998). Researchers refer to this phenomenon as “developmentally limited alcoholism” (Zucker, 1987) or “maturing out” of problem drinking (Donovan, Jessor, & Jessor, 1983; Miller-Tutzauer, Leonard, & Windle, 1991; O'Malley, 2004-2005). Despite this phenomenon, however, college students' excessive alcohol consumption poses a very real threat to their safety and welfare. For example, the heavy drinking that occurs during the college years places students (both those who do, and do not, “mature out” of problem drinking) at risk for numerous deleterious and potentially life-altering consequences such as uninten-tional injury, engaging in unprotected sexual intercourse, violence, assault, rape, and alcohol-related motor vehicle crashes (Hingson, Heeren, Zakocs, Kopstein, & Wechsler, 2002; Hingson, Heeren, Winter, & Wechsler, 2005). Additionally, the college student population has the greatest percentage of problem drinkers (as defined by DSM-IV alcohol dependence), when compared to other demographic age groups (NIAAA, 2008).

While it is possible for students to transition out of their excessive drinking behaviors *after* college, it is imperative that college students avoid succumbing to the harmful alcohol-related consequences associated with their drinking patterns during college. In fact, college students themselves recognize the need for such educational/interventional programming aimed at reducing negative alcohol-related consequences. For instance, after conducting a qualitative investigation into the methods college students employ to minimize alcohol consumption-related harm to both themselves and others, Howard, Griffn, Boekeloo, Lake, and Bellows (2007) concluded, “In terms of informational and behavioral needs, students expressed both frustration at being taught only to abstain from drinking and genuine interest in acquiring Specific kinds of knowledge and skills. Salient among their concerns was knowing how to *drink responsibly* [emphasis added]…” (p. 252).

While researchers have attempted to include responsible drinking as a behavioral outcome in their interventions, so far these attempts have suffered from serious methodological limitations. Specifically, researchers are “consistently inconsistent” in their efforts to identify explicit characteristics of responsible drinking (Barry & Goodson, 2010, p. 301). To date, there is a dearth of both evidence-based and theoretically derived research identifying Specific, empirical, responsible drinking characteristics (Barry & Goodson, 2010). Thus, attempting to instruct college students (or anyone else) in Specific responsible drinking practices becomes equivalent to building a house on sand: the foundation is not securely anchored, the ground shifts repeatedly, and the structure lacks stability. Put simply, prior to developing responsible drinking interventional and/or education programming, it is important to first establish the contextual factors which may influence one's responsible drinking practices. Once established, these factors will provide researchers and practitioners with valuable insight into (a) the factors that facilitate responsible drinking and (b) the barriers inhibiting responsible drinking practices.

Although an initial investigation into the Specific beliefs and behaviors college students associate with responsible drinking has been conducted (Barry & Goodson, 2011a), to date, there is no substantive research establishing the various contextual factors that may influence the practice of these beliefs. Consequently, this article seeks to expand the currently limited body of evidence associated with responsible drinking by reporting (a) the contextual factors infuencing one's decision to drink responsibly, (b) the prevalence of these factors within a sample of Texas college students, and (c) whether the prevalence of these factors varies as a function of sex and/or binge drinking status.

As a caveat, we wish to point out that this study does not address “moderate drinking” (Dufour, 1999; Green, Polen, Janoff, Castleton, & Perrin, 2007), a construct sometimes associated with responsible drinking. Instead, exclusive focus was devoted to responsible drinking and the contextual factors that influence its practice. Some might argue that responsible drinking closely relates to moderate drinking, but we contend that systematic examination of responsible drinking must take place before it can be subsumed within an already defined construct [up to one drink per day for women and two drinks per day for men (USDHHS & USDA, 2000)]. Furthermore, previous investigations into the beliefs and behaviors college students associate with responsible drinking document moderate drinking as only one of the many themes associated with responsible drinking; thus, moderate drinking is not the overarching construct enveloping the conceptualization and practice of responsible drinking (Barry & Goodson, 2011a).

## METHODS

This study employed a partially mixed sequential dominant status design (Leech & Onwuegbuzie, 2009), or a mixed methods design unfolding in two phases. This design (usually denoted by the abbreviation “qual → QUAN”) organizes the study in two sequentially occurring phases, with an emphasis being placed on the latter, quantitative phase. Creswell, Plano Clark, Gutmann, and Hanson (2003) contend that this strategy is best suited for exploring a phenomenon in which there is no guiding framework/theory. Considering the limited scope of the published literature associated with responsible drinking, this methodology is appropriate. Procedures for both phases of this investigation were vetted, and approved, by the Institutional Review Board (IRB) where the samples were recruited.

### Phase One—Qualitative

The initial phase of this investigation sought to qualitatively explore the contextual factors infuencing one's responsible drinking practices. Due to the dearth of systematic, published investigations into responsible drinking, this phase encompassed a series of less structured focus group sessions. Less structured groups are an ideal choice when researchers do not have prior knowledge/insight into the topic they are investigating (Morgan, 1998). An “emergent design“ approach also guided the data collection process (Lincoln & Guba, 1985).

*Participants*. Participants were recruited from several health promotion core-content courses offered at a large, four-year public university in Texas. The first author visited randomly chosen class sessions to provide information regarding the purpose and overall objectives of the study. Once informed about the objectives, students could indicate their interest in participating in the study, by providing (on an index card) their name, e-mail address, and the most convenient day(s) and time(s) for meeting with a focus group. The final sample size comprised 13 individuals, two men and 11 women. The majority of the sample was Caucasian (*n* = 11), with nominal representation of African-Americans (*n* = 1; female), and Hispanics (*n* = 1; male).

*Data Collection*. Prior to beginning each focus group session, participants reviewed and signed an IRB-approved informed consent form. As outlined in the consent document, each session was audio-taped. In total, four focus group sessions and three individual interviews (necessary in order to accommodate participants' scheduling conflicts) were conducted. The first author facilitated each focus group session and conducted each interview. During focus group or interview sessions, participants were asked to discuss the contextual factors infuencing their responsible drinking behaviors. Specifically, participants were asked a series of questions including, but not limited to, “Can you think of any barriers and/or obstacles that would prevent you or your peers from drinking re-sponsibly?”, “What types of situations would impact your ability to drink responsibly?”, “Can you think of a situation in which it would be impossible for you or someone else to drink responsibly?”, and “Can you provide some examples of how you could drink in a more responsible manner?” During discussions, participants were probed to provide Specific norms, attitudes, and/or beliefs associated with their contributions. All identifying characteristics or personal descriptions (i.e., name, age, etc.) were removed from the typed transcripts as well as from any presented or published accounts of the sessions, to ensure confidentiality.

*Data Analysis*. At the conclusion of each session, typed transcripts were developed from the audio-recordings. Using the constant comparison model (Lincoln & Guba, 1985) to sort and classify recurrent or significant themes within participants' responses, we identifed (highlighted the text) and extricated (placed the text into an unconnected document) each distinct idea/thought from all transcripts. Each idea/thought unit was then grouped with similar ones, and each group of similar ideas formed a category and received a label. Each labeled category became an overarching theme. This particular method of thematic analysis has also been referred to as a general inductive approach (Thomas, 2006).

(1) *FINDINGS: Contextual Factors Infuencing Responsible Drinking*. Overall, the emerging contextual factors infuencing the practice of responsible drinking were either intrapersonal (i.e., internal) or environmental (i.e., external) in nature. Grounded in the Ecological Perspective (McLeroy, Bibeau, Steckler, & Glanz, 1988), intrapersonal factors comprise a person's cognitive, affective, or behavioral traits/characteristics that influence behavior, such as knowledge, attitudes, and beliefs. The intrapersonal factors emerging from the focus groups and interview sessions, deemed to influence one's decision to drink responsibly, centered on “personal responsibilities” and “emotional status” at the time of drinking. Environmental factors infuencing responsible drinking, on the other hand, comprised interactions between family, friends, and peers as well as social networks and/or norms associated with group membership. Specific environmental factors emerging from the focus groups and interview sessions included subthemes of “surrounding environment,” “monetary considerations,” “drinking games,” and “other people.”
(2) *Intrapersonal Factors: Personal Responsibilities*. Participants believed their school-related obligations represented a salient factor impacting their responsible drinking practices. For example, the following days' school schedule emerged as a factor affecting decision-making: “Do I have class at eight in the morning? If so, maybe I shouldn't stay out until four O'clock.” Another participant echoed the impact of school-related factors, saying:
  You may go somewhere [social environment, bar, etc.] knowing you have a test the next day and say “I am just going to go for a little bit. I will just have one or two drinks.” But then if you have one or two drinks, you might come home and be tired, not study for that test like you were supposed to, and do poorly.
  In addition to school-related factors, scenarios involving personal accountability and responsibility also emerged. One such example was the health of an unborn baby, or “not drinking during pregnancy.” This participant elaborated on the manner in which her motherly duties influenced her drinking behaviors, stating:
  I know that if I go somewhere and I drink and then I have to come home and take care of my kids I know I cannot do it well if I have had drinks, or maybe the next morning I will not be able to be as attentive as I would have been if I didn't have drinks.

Thus, personal responsibilities, whether school or family-related, emerged as an intrapersonal factor infuencing one's decision to drink responsibly.

(1) *Emotional Status*. An individual's emotional state/status also emerged as a factor potentially impacting responsible drinking behavior. This subtheme revolved around feelings, such as anxiety, stress, or depression. For example, one participant stated, “sometimes you are feeling depressed or stressed out and you want to get away from that, and that causes people to drink more than they usually would.” Participants cited Specific, hypothetical, instances that could impact one's decision to drink responsibly. Some of these instances inhibiting ability and/or decision to drink in a responsible manner, ranged from work-related circumstances [“a bad day at work”] to relationship problems [“boyfriend or girlfriend broke up with them”].
(2) *Environmental Factors: Monetary Considerations*. Participants felt carrying an excess of cash while in a social setting, such as a bar, could influence drinking behavior. Specifically, excess money allowed additional drinks, should he/she feel the urge, to be purchased at whim. As a preventative measure, and strategy to limit consumption, participants allocated a set amount of money to an evening's-worth of alcohol. Participants felt this strategy prevented them from giving in to temptation and assisted in limiting alcohol consumption. In other words, persons used money as a meter for determining how much had been (and could be) consumed; once the money was gone, drinking stopped. For instance, one participant revealed “I will only bring enough money for a certain amount of drinks. If you cannot afford to have more, you aren't tempted to drink more because you don't have the money for it.” Another participant echoed this sentiment, stating “I make sure not to bring a bunch of money to the bar that I could spend on drinks.” In addition to applying this concept in a social setting, others implemented this strategy when purchasing alcohol for consumption at home: “I only like to buy a certain amount because I will know exactly how much I am going to drink. That makes me feel very comfortable.” When probed to develop this point, the participant offered the following analogy: “It is like cooking extra; if you don't cook extra food then you will not eat more.” Similarly to cash, the utilization of credit cards emerged as an influence on responsible drinking. Specifically, the “open tabs” that credit cards allow was characterized as a detriment to responsible drinking behavior. By keeping an “open tab,” participants felt it was easy to lose track of both the amount spent on alcohol, as well as the number of drinks consumed. For these reasons, one participant identifed credit card use at a bar as “terrible.” This individual elaborated, stating “my friends get open tabs with their credit cards and by the end of the night they are like ‘wow, I did not realize this!”’
(3) *Surrounding Environment*. Another external factor potentially impacting responsible drinking included the surrounding environment. This notion applied not only to the location where drinking occurs (physical environment), but also to the individuals present at the time (social environment). With regard to the physical environment, participants felt one needs to “be in a safe setting.” Participants depicted a safe environment as, “go to someone's house to drink all night and stay the entire night there.” In addition to considering where one will drink, participants insisted the individuals present should also be considered. As one person stated, “If you are going to drink alcohol you should feel comfortable and trust people you are around.” Thus, participants felt it was important to consider not only where one will be drinking, but also who is present and how well they're known. One participant asserted,
  You need to make sure that you are around people you are familiar with. You have to trust the people you are around. If you are just in a bar getting drunk, you do not know the people you come into contact with.
  Elaborating on the importance of one's drinking environment, and those in it, multiple female participants highlighted the dangers of leaving a drink unattended: “Watch your drink. Do not set them down because people could always put something in them.” Multiple female participants were wary of other individuals and fearful of being dosed with a “date rape drug.” These admissions clearly point to a gender-related concern, as none of the males in the current sample discussed or alluded to consequences associated with leaving a drink unattended.
(4) *Drinking Games*. The current sample also identifed participation in drinking games as precluding drinking in a responsible manner. Borsari (2004) asserts, “Drinking games often stimulate a competitive environment, replete with winners, losers and spectators” (p. 37). One participant echoed this sentiment, saying participation in a drinking game was “the same as if you were in sports or an athletic competition.” The participant elaborated, “this person says that they can do this many shots, well then I have to one up that. I cannot let that person out show me.”
(5) *Other People*. Lastly, other persons were also cited as impacting one's decision to drink responsibly. One form of influence alluded to in our sample was peer pressure. Specifically, participants provided insight into two distinct types of peer pressure infuencing responsible drinking: indirect and direct. Borsari and Carey (2001) characterize these separate, Specific types of peer pressure in a review outlining the peer in-fuences on college alcohol use, stating: “Direct (or active) peer influences explicitly focus on getting a person to drink, and can range from polite gestures (e.g., offering to get a peer a drink, buying a round) to overt commands or encouragement to drink (e.g., forcing others to drink during drinking games)” (p. 393). Indirect peer pressure, however, includes providing “information about what behaviors are accepted and admired, what is considered appropriate in a given social context, and therefore what behaviors are likely to lead to social acceptance and reinforcement” (p. 393).

Instances exemplifying direct peer pressure among our sample were primarily related to hazing. However, the apparent pressure applied on participants was not restricted to brute force. For instance, the simple gesture of offering a drink could have profound influence: “I think a single girl just going to the bar with her friends to have fun and plans on not drinking, then a cute guy buys her a drink, I think that she would probably take it.” Thus, whether being propositioned or forced to consume alcohol, one's ability to drink responsibly was cited as being hindered by direct peer pressure. Examples of indirect peer pressure primarily stemmed from social norms and group membership. One participant explained, “if all your friends' activities revolve around drinking, then it becomes part of hanging out. You don't want to miss out on time with your friends so you drink too.” Said differently, “If everyone is at the party and everyone is drinking then you might feel left out.”

A subtheme associated with the influence others exert on responsible drinking behavior emerged in the concept of a “designated caretaker.” Participants in our study defined this drinking “buddy-system,” as having another individual, whom the drinker trusts, make decisions for the drinker while he/she is intoxicated. This practice was identifed as a method not only to ensure the drinker's safety, but also to accomplish responsible drinking. As one participant explained, a caretaker is “there to watch out for you and make sure that you are going to have a safe way to get home and that no one can take advantage of you.” At its core, this theme centered on the concept of having a known, trusted friend who could “make wise choices about any situation that may arise that night.” A participant-provided analogy equated a designated caretaker to “a sort of mother hen.”

### Phase Two—Quantitative

At the conclusion of Phase One, the aforementioned themes were used as the foundation for the development of two quantitative scales. In other words, based upon the participant-provided contributions, a set of items was developed to address the contextual themes emerging from Phase One. Once developed, these scales allowed for the second phase of this study to be completed. Specifically, we (a) assessed the prevalence of the contextual factors among a sample of college students attending a large, Texas, public institution, and (b) determined whether the prevalence of these factors varied as a function of both sex and alcohol consumption.

*Sample*. A randomly selected sample of currently enrolled students was solicited via e-mail to participate. Of the 5,000 invitations sent, 4,985 were received. Of those successfully contacted, a total of 729 (15% response rate) students returned usable surveys. On average, respondents were 22 years of age (*SD* = 5.49). Females (55%) represented a slight majority in this sample. Ethnically, the majority of the sample was Caucasian (76%); while the remainder were Hispanics (10%), Asian or Pacifc Islander (7%), African-American (2%), Eastern Indian (1%), American Indian or Alaskan Native (0.5%), Middle Eastern (.5%), or Other (2.3%). That said, it is important to note that the demographic (gender and ethnic) distribution of this sample is comparable to the population from which it was drawn. While primarily Caucasian (73%), the university's three largest ethnic groups are also Hispanics (11%), Asians (4%), and African-Americans (3%). Females represent 47% of the institution's student body and were, therefore, slightly over-represented in our sample. Considering the low proportion of individuals in ethnic backgrounds other than Caucasian and Hispanic, for data analysis purposes all non-Caucasian and non-Hispanic ethnicities were collapsed into an “Other” category in order to ensure enough participants were included in each ethnic group.

*Measures*. To assess the prevalence of contextual factors infuencing college students' responsible drinking practices, two distinct scales were employed. All included scale items were developed based upon the themes emerging from the qualitative data from Phase One. For example, on the responsible drinking motivations scale (21 items), respondents were asked to indicate if work- or school-related obligations, monetary concerns, or the actions of others would motivate drinking responsibly. Additionally, as part of the responsible drinking barriers scale (16 items), respondents indicated if being challenged to a drinking contest, peer pressure (both direct and indirect), or performing badly on school assignments would be a barrier to drinking responsibly. For each item in the scales utilized, respondents were asked to indicate whether the given conditions/situations served as a potential motivator/barrier to drinking responsibly (1) never, (2) seldom, (3) some of the time, (4) most of the time, or (5) always. See Tables 1 and 2 for Specific wording of each item and its associated response scale.

**Table 1:** Responsible drinking motives across genders

CHORDS motivations scale items				

When I drink responsibly, one of my motives is…	Male	Female	p	Cohen's d
1. Because I do not want to get drunk.	3.44	3.60	.095	–
2. Because I have to look out for one of my friends.	3.34	3.45	.209	–
3. Because of my religious convictions.	2.16	2.43	.033	–0.189
4. Because I do not want to do anything out of my character I may later regret.	3.37	3.90	.000	–0.440
5. Because I do not want to spend a lot of money on alcohol.	3.22	3.06	.128	–
6. Because my significant other or parent(s) will be upset with me if I drink too much	2.64	2.50	.187	–
7. Because I have to drive myself home.	3.65	3.75	.338	–
8. Because I do not want someone to take advantage of me.	1.97	3.50	.000	–1.161
9. Because I am afraid of getting in trouble with law enforcement.	3.24	3.46	.066	–
10. Because I do not want to develop a drinking problem.	2.46	2.48	.924	–
11. Because I want to have control over my actions.	3.65	4.07	.000	–0.379
12. Because of my work-related responsibilities.	2.77	2.74	.827	–
13. Because I am the designated driver.	3.44	3.63	.100	–
14. Because I do not want to get nauseous or vomit.	3.33	3.69	.001	–0.282
15. Because I want to be aware of and understand what is going on around me.	3.54	3.89	.000	–0.311
16. Because I have to get up early in the morning for class.	3.17	3.19	.862	–
17. Because a friend and/or family member hasadrinking problem.	1.94	2.16	.066	–
18. Because I want to remember what happens.	3.07	3.57	.000	–0.389
19. Because I need to study foratest or complete my school work.	3.26	3.29	.792	–
20. Because I want to keep my blood alcohol concentration (BAC) under 0.08%.	2.16	2.41	.027	–0.196
21. Because I am with people I do not know very well or in a new environment.	2.62	2.98	.001	–0.300

Response Scale: (1) Never a motivator for responsible drinking, (2) Seldom a motivator for responsible drinking, (3) A motivator for responsible drinking some of the time, (4) A motivator for responsible drinking most of the time, or (5) Always a motivator for responsible drinking.

**Table 2:** Responsible drinking barriers across genders

CHORDS barriers scale items				

The next time I drink alcohol, I would not be able to drink responsibly if…	Male	Female	p	Cohen's d
1. I felt depressed or stressed out.	2.13	2.32	.071	–
2. I had recently failed an important test in one of my classes.	2.05	2.09	.699	–
3. I had recently broken up with my significant other.	2.36	2.44	.503	–
4. Everyone else was getting drunk.	2.34	2.37	.811	–
5. I had a designated driver.	2.40	2.39	.887	–
6. An attractive person wanted to buy meadrink(s).	2.46	2.08	.001	0.302
7. I was having a bad day.	2.21	2.29	.414	–
8. I was playing a drinking game.	2.97	2.71	.036	0.189
9. I felt like I would be missing out on a good time with my friends.	2.57	2.38	.067	–
10. I was an alcoholic.	2.17	1.92	.089	–
11. I was celebrating my 21st birthday.	3.08	3.11	.886	–
12. I had someone challenge me to a drinking contest.	2.20	1.86	.001	0.294
13. I felt pressured by friends to drink.	2.07	1.89	.053	0.174
14. I was at a party and/or friend's house and planned on staying there that night.	2.86	2.79	.534	–
15. Someone I trust agreed to stay sober to look after me and make sureI was safe.	2.46	2.53	.499	–
16. I had a family member that has a drinking problem.	1.61	1.56	.540	–

Response Scale: (1) Never an obstacle to drinking responsibly, (2) Seldom an obstacle to drinking responsibly, (3) An obstacle to drinking responsibly some of the time, (4) An obstacle to drinking responsibly most of the time, or (5) Always an obstacle to drinking responsibly.

Before analyzing the data produced from these scales, the internal consistency (i.e., reliability) of the motivation and barriers scales employed in this investigation were evaluated. With this study's sample, the motivation scale exhibited a Cronbach coeffcient alpha of 0.87 and the barriers scale, a 0.91. Scales exhibiting coeffcient alphas within this range have been deemed “very good” (DeVellis, 2003). Both scales also exhibited strong score validity. Specifically, a principal component factor analysis (PCA) revealed the 21 items of the motivation scale accounted for 57.7% of the total variance, while the barrier scale's 16 items accounted for 62.2% of the variance.

*Data Collection*. In order to obtain a representative sample, the investigators obtained a list of all currently enrolled undergraduate and graduate students attending the institution. This list contained students' full names and their corresponding e-mail addresses. Using the list as a sampling frame, a random sample was invited to participate in the study via e-mail. Contacted individuals had approximately seven days to complete the online survey before the link would become inactive. Reminders were sent to those who had not yet accessed or completed the survey on days three and five. In order to access the survey, individuals clicked on a hyperlink embedded within the invitation and/or reminder e-mail. Persons clinking on the weblink were subsequently directed to an Information Sheet outlining informed consent matters. A “Go to Survey” link was located at the bottom of the information sheet. By clicking on the “Go to Survey” link, participants were confrming their understanding and voluntary acceptance of the study procedures.

*Data Analysis*. Prior to data analysis, participant non-response was examined. After taking into account non-response due to embedded skip patterns (e.g., abstained from alcohol consumption), all motivation scale items exhibited less than 1% missing data and all barriers scale items exhibited no more than 5%. Thus, the quantity of missing data for the measures was quite low. With regard to the pattern of missing data, nonresponse on both the motivation and barrier scales were examined. Independent sample *t*-tests confrm that respondents with fully completed motivation scales did not significantly differ from those with missing responses with regard to gender [*t*(689) = −0.530, *p* < .107] or binge drinking status [*t*(687) = 0.302, *p* < .510]. Similarly, those who fully completed all the barrier scale items did not significantly differ from respondents with missing responses based on gender [*t*(689) = −0.193, *p* < .650] or binge drinking status [*t*(687) = −0.426, *p* < .447]. Due to the lack of large amounts of missing data, as well as the manner in which data were missing, it could not be justifed to employ complex analytical technique(s) to calculate imputable scores. Moreover, it was determined that data were missing at random (MAR). Researchers categorize data MAR as “ignorable” (Buhi, Goodson, & Neliands, 2008). Consequently, incomplete surveys were retained for data analysis and missing data were deleted (listwise) from the analysis.

Utilizing the Predictive Analytics SoftWare (PASW) (version 18.0), basic descriptive statistics (Mean ± SD) were generated for each item on the two included scales. Sex- and consumption-based differences for each scale were calculated using one-way ANOVA. Cohen's *d* effect sizes were calculated for all statistically significant ANOVA fndings in order to further understand the strength of the observed relationship. Lastly, we employed logistic regression to assess the strength of the association among one's responsible drinking motivations, barriers, and reported drinking behavior. Age, sex, and race served as covariates for the logistic regression analysis.

*Results*. In establishing the extent to which a factor served to facilitate or impede responsible drinking practices, there was a clear distinction between male and female responses across the motivations and barriers scales. Females were more likely to indicate that an item on the motivations scale facilitated responsible drinking, while less likely to indicate that an item on the barriers scale impeded responsible drinking. In other words, males identifed more factors as obstructing responsible drinking practices, while at the same time identifying fewer factors aiding drinking responsibly.

Women were significantly more likely to identify the following factors as a motivator to responsible drinking: When I drink responsibly, one of my motivations is… “because of my religious convictions” [*F*(1,513) = 4.546, *p* < .033]; “because I do not want to do anything out of my character I may later regret” [*F*(1,512) = 25.109, *p* < .0001]; “because I do not want someone to take advantage of me” [*F*1(1,512) = 170.784, *p* < .0001]; “because I want to have control over my actions” [*F*(1,513) = 18.643, *p* < .0001]; “because I do not want to get nauseous or vomit” [*F*(1,512) = 10.217, *p* < .001]; “because I want to be aware of and understand what is going on around me” [*F*(1,511) = 12.529, *p* < .0001]; “because I want to remember what happens” [*F*(1,513) = 19.489, *p* < .0001]; “because I want to keep my blood alcohol concentration (BAC) under 0.08%” [*F*(1,514) = 4.912, *p* < .027]; and “because I am with people I do not know very well or in a new environment” [*F*(1,512) = 11.444, *p* < .001]. See Table 1 for mean male and female responses to the motivations scale items as well as gender-based differences.

Regarding factors serving as barriers to responsible drinking, women were significantly less likely to identify the following factors as barriers to responsible drinking: The next time I drink alcohol, I would not be able to drink responsibly if… “an attractive person wanted to buy me a drink(s)” [*F*(1,491) = 11.393, *p* < .001], “I was playing a drinking game” [*F*(1,494) = 4.411, *p* < .036], “I had someone challenge me to a drinking contest” [*F*(1,491) = 10.726, *p* < .001], and “I felt pressured by friends to drink” [*F*(1,491) = 3.752, *p* < .05]. See Table 2 for mean male and female responses to the barriers scale as well as gender-based differences. Effect sizes (Cohen's *d*) are reported in conjunction with all significant group differences presented in both Tables 1 and 2. To interpret this index, Cohen (1988) supplies the following categorizations: small (.20), moderate (.50), and large (.80). While these designations have been criticized, these categories do provide insight into the degree to which the null hypothesis is false,

ANOVAs were also conducted to determine whether factors facilitating or impeding responsible drinking varied as a function of one's alcohol consumption (i.e., binge drinking status). Respondents were asked to respond to the following question “Think back over the last two weeks. How many times, if any, have you had fve or more alcohol drinks at a sitting?” Participant responses ranged from none (65.2%), to one time (13.2%), two times (8.0%), three times (5.5%), four times (3.3%), fve times (1.6%), six times (1.9%), seven times (0.3%), eight times (0.1%), and nine or more times (0.9%). In order to group respondents based upon binge drinking status, responses were dummy coded as “0” for no times within the past two weeks, and “l” for one or more times within the past two weeks. After grouping respondents based upon their binge drinking behaviors, the majority of the items for both the motivations and barriers scales now violated Levene's statistic (an underlying assumption of homogeneity of variance). Consequently, we employed Brown–Forsythe's robust test of equality of means to account for the statistically significant Levene's statistic. Thus, the *p*-values outlined in both Tables 3 and 4 are based upon the Brown–Forsythe test.

**Table 3:** Responsible drinking motives as a function of binge drinking

CHORDS motivations scale items				

When I drink responsibly, one of my motives is…	Non-binge Binge drinker	Binge drinker*	p**	Cohen's d
1. Because I do not want to get drunk.	3.91	3.04	.001	0.837
2. Because I have to look out for one of my friends.	3.49	3.29	.016	0.212
3. Because of my religious convictions.	2.66	1.83	.001	0.613
4. Because I do not want to do anything out of my character I may later regret.	4.01	3.22	.001	0.678
5. Because I do not want to spend a lot of money on alcohol.	3.28	2.95	.002	0.281
6. Because my significant other or parent(s) will be upset with me if I drink too much	2.73	2.35	.001	0.303
7. Because I have to drive myself home.	3.79	3.61	.092	–
8. Because I do not want someone to take advantage of me.	3.11	2.43	.001	0.456
9. Because I am afraid of getting in trouble with law enforcement.	3.45	3.25	.094	–
10. Because I do not want to develop a drinking problem.	2.72	2.16	.001	0.387
11. Because I want to have control over my actions.	4.23	3.43	.001	0.771
12. Because of my work-related responsibilities.	2.82	2.67	.186	–
13. Because I am the designated driver.	3.59	3.52	.549	–
14. Because I do not want to get nauseous or vomit.	3.75	3.25	.001	0.397
15. Because I want to be aware of and understand what is going on around me.	4.06	3.31	.001	0.723
16. Because I have to get up early in the morning for class.	3.15	3.22	.468	–
17. Because a friend and/or family member has a drinking problem.	2.21	1.85	.002	0.274
18. Because I want to remember what happens.	3.60	3.01	.001	0.469
19. Because I need to study for a test or complete my school work.	3.12	3.49	.001	–0.305
20. Because I want to keep my blood alcohol concentration (BAC) under 0.08%.	2.64	1.86	.001	0.631
21. Because I am with people I do not know very well or in a new environment.	2.95	2.64	.003	0.261

* Respondent binge drinking status determined by self-reporting consuming “fve or more drinking at a sitting” within the last two weeks one or more times.

** In order to account for unequal variance, statistical signifcance based upon Brown–Forsythe's robust test of equality of means.

**Table 4:** Responsible drinking barriers as a function of binge drinkings

CHORDS barriers scale items				

The next time I drink alcohol, I would not be able to drink responsibly if…	Non-binge drinker	Binge drinker*	p**	Cohen's d
1. I felt depressed or stressed out.	2.09	2.41	.002	–0.283
2. I had recently failed an important test in one of my classes.	1.87	2.34	.001	–0.423
3. I had recently broken up with my significant other.	2.13	2.77	.001	–0.493
4. Everyone else was getting drunk.	2.08	2.72	.001	–0.567
5. I had a designated driver.	2.03	2.89	.001	–0.683
6. An attractive person wanted to buy meadrink(s).	1.95	2.68	.001	–0.590
7. I was havingabad day.	2.02	2.56	.001	–0.504
8. I was playing a drinking game.	2.42	3.39	.001	–0.751
9. I felt like I would be missing out on a good time with my friends.	2.17	2.85	.001	–0.607
10. I was an alcoholic.	1.97	2.13	.282	–
11. I was celebrating my 21st birthday.	2.64	3.69	.001	–0.703
12. I had someone challenge me to a drinking contest.	1.71	2.43	.001	–0.629
13. I felt pressured by friends to drink.	1.88	2.10	.023	–
14. I was at a party and/or friend's house and planned on staying there that night.	2.50	3.25	.001	–0.623
15. Someone I trust agreed to stay sober to look after me and make sure I was safe.	2.21	2.88	.001	–0.545
16. I had a family member that has a drinking problem.	1.53	1.64	.260	–

* Respondent binge drinking status determined by self-reporting consuming “fve or more drinking at a sitting” within the last two weeks one or more times.

** In order to account for unequal variance, statistical signifcance based upon Brown–Forsythe's robust test of equality of means.

Those who had consumed fve or more drinks in one sitting at least once within the past two weeks also exhibited significantly different motivators to responsible drinking. Specifically, binge drinkers nearly universally reported each of the specifed factors would be less of a motive for drinking responsibly than nonbinge drinkers. In other words, nonbinge drinkers acknowledged more motives to drink responsibly than binge drinkers. Of the 21 items included in the scale, there was a statistically significant group difference for 16 of them. Effect size relationships ranged from 0.21 (small—When I drink respon-sibly one of my motive is because I have to look out for one of my friends) to 0.83 (large—When I drink responsibly one of my motive is because I do not want to get drunk). See Table 3 for mean responses of binge drinkers and nonbinge drinkers and effect sizes.

While binge drinkers reported nearly all the motives scale factors as less likely to facilitate responsible drinking, they also identifed the vast majority of items on the barriers scale as factors inhibiting responsible drinking. Thus, binge drinkers identifed more barriers to drinking responsibly when compared to their nonbinge drinking peers. Binge drinkers were significantly different from nonbinge drinkers in 13 of the 16 items on the barriers scale. Specifically, binge drinkers identifed all but one factor on the barriers scale as an impediment to responsible drinking, compared to their nonbinge drinking peers. Effect size relationships ranged from 0.28 (small—I would not be able to drink responsibly if I felt depressed or stressed out) to 0.75 (large—I would not be able to drink responsibly if I was playing a drinking game). See Table 4 for mean responses of binge drinkers and nonbinge drinkers and effect sizes.

In order to assess the strength of the association among one's responsible drinking motivations, barriers, and reported drinking behavior, we conducted a multivariate logistic regression analysis. Specifically, we sought to determine whether one's composite score on the motivations and barriers scales (independent variables) would be associated with individuals' binge drinking behavior within the past two weeks (dependent variable), after controlling for sex, age, and ethnicity. Prior to the analysis, multicollinearity was assessed to determine if any of the independent variables and covariates were highly correlated. Since the highest Pearson correlation between variables was 0.199, it was concluded that collinearity would not influence the logistic regression analysis. Overall, the full model was statistically significant (χ^2^ with 6 *df* = 99.422, *p* < .0001), indicating the model was able to distinguish between those who had, and had not, binged within the past two weeks. Further supporting the model's goodness-of-fit and overall reliability, the Hosmer and Lemeshow test was not significant (χ^2^ with 8 *df* = 10.803, *p* < .213). In all, the model as a whole explained between 20.8% (Cox & Snell *R*^2^) and 27.9% (Nagelkerke *R*^2^) of the variability associated with one's binge drinking status, and correctly classifed 71.2% of cases, an increase of 14% over the original model. As shown in Table 5, even when controlling for the covariates of age, sex, and ethnicity, the motivations scale (OR = .396, Wald = 22.044, *df* = 1, *p* < .0001) and barriers scale (OR = 2.548, Wald = 39.382, *df* = 1, *p* < .0001) exhibited strong associations with binge drinking status. Participants more motivated to drink responsibly were less likely to have engaged in binge drinking within the past two weeks (*B* = −0.926), while participants who perceived more barriers to drinking responsibly were more likely to have engaged in binging (*B* = 0.935). In addition to being Hispanic (OR = 3.423, Wald = 5.201, *df* = 1, *p* < .023), age was also significantly related to binge drinking status (OR = 0.944, Wald = 4.840, *df* = 1, *p* < .028): younger participants (B = −0.058) were more likely to have binged in the weeks prior to the survey.

**Table 5:** Logistic regression predicting binge drinking status

	95% C.I. for odds ratio							
	
	Odds ratio	Lower	Upper	B	S.E.	Wald	df	Sig.
Motivations scale composite	0.396	0.269	0.583	–0.926	0.197	22.044	1	0.0001
Barriers scale composite	2.548	1.903	3.413	0.935	0.149	39.382	1	0.0001
Age	0.944	0.896	0.994	–0.058	0.026	4.840	1	0.028
Sex	0.673	0.429	1.055	–0.397	0.230	2.976	1	0.085
			Race					
White non-Hispanic	1.454	0.633	3.343	0.374	0.425	0.777	1	0.378
Hispanic or Latino	3.423	1.189	9.854	1.230	0.540	5.201	1	0.023
Constant	4.050	–	–	1.399	1.031	1.842	1	0.175

In order to further assess the independence of the motivations and barriers scales, with regard to their association with binge drinking, a separate model was tested. Specifically, we conducted a subsequent multivariate logistic regression analysis that excluded the barriers scale, to determine the amount of shared variance across the two scales and their association to binge drinking status. Without the barrier scale included, the motivations scale (*B* = −1.041) association to binge drinking remained statistically significant (OR = 0.353, Wald = 35.022, *p* < .0001); however, changes in the odds ratio were minimal. Thus, while both the motivations and barriers scales are clearly associated with one's binge drinking, these two factors account for different aspects of this behavior.

## DISCUSSION

The initial phase of this investigation qualitatively explored the contextual factors infuencing the practice of responsible drinking. As a whole, the participants clearly articulated several motivating and inhibiting factors infuencing one's ability to drink responsibly. Due to the college student-status of the participants, a number of these factors seemed uniquely tied to their collegiate status and/or experiences. For instance, school-related obligations, such as class and/or a test, emerged as a predominant aspect of their lives (e.g., “When I drink responsibly, one of my motives is because I need to study for a test or complete my school work”—See Table 3). As such, it is conceivable that in a similar fashion, work-related responsibilities would emerge as a factor in research examining younger and older adults not enrolled in a college or university. Nevertheless, an increasing number of studies document the positive influence next-day responsibilities (e.g., class, test, etc.) have on the drinking levels of college students (Correia, Benson, & Carey, 2005; Skidmore & Murphy, 2011; Wood, Sher, & Rutledge, 2007). For example, even after controlling for GPA and high school alcohol consumption, college students who do not have Friday morning classes drink two times as much alcohol on Thursday evenings than those students who do have Friday morning classes (Sher & Rutledge, 2007). Classes prior to 10:00 am Friday morning were found to have the greatest influence on prior night drinking. In other words, “Next-day day classes or tests can be viewed as either an alternative reinforcer or as an indirect means of increasing the real cost of drinking” (Skidmore & Murphy, 2011, p. 65). Results from the current investigation (both qualitative and quantitative) echo the protective effect school-related responsibilities can have on the drinking behaviors of college students.

The impact of the collegiate experience on responsible drinking also emerged in the form of drinking games (i.e., drinking competitions). While previous research clearly indicate that drinking games significantly contribute to heavy drinking (Borsari, 2004), the growing literature base associated with drinking games exclusively examines the college population. As indicated in their titles, these games foster a sense of competition among participants. Therefore, not only does competition foster drinking at an increased rate, but it also encourages consuming a greater amount of alcohol in an effort not to be one-upped by another person. Johnson & Sheets (2004) conclude “available evidence suggests that drinking games are associated with greater or more rapid consumption of alcohol than in other contexts” (p. 91). As evident in the contributions of participants, responsible drinking would be diffcult to accomplish if one found him/herself in a drinking competition or situation that called for consuming large amounts of alcohol in order to demonstrate dominance. Moreover, our quantitative results document being challenged to a drinking contest and/or participating in a drinking game as highly significant barriers to responsible drinking practices (See Table 4).

Among the current sample, financial considerations were also found to influence the practice of responsible drinking behavior. In addition to preventing excessive consumption by only allocating a set amount of money for the purchase of drinks, limiting money spent on alcoholic beverages was a significant motivator for responsible drinking. These findings parallel previous research documenting the infuential role of the costs of alcohol on drinking quantity. Specifically, drinking level decreases among college students as the price of alcohol increases (Kuo, Wechsler, Greenberg, & Lee, 2003; Murphy & MacKillop, 2006; Skidmore & Murphy, 2011). Moreover, even nominal increases in the price of alcohol at on-premise establishments have been associated with decreases in patron intoxication level when leaving a bar (O'Mara et al., 2009). Thus, stricter regulation on the price and discounting of alcohol may also lead to increased practice of responsible drinking practices.

Peer pressure also emerged as a dominant force on one's responsible drinking behaviors. This theme was not surprising, given that scholarly reports consistently note the indelible influence peers have on the development and maintenance of drinking behaviors among college students (Borsari & Carey, 2001). For example, researchers note peer drinking as a significant predictor of alcohol misuse among adolescents (Tyler, Stone, & Bersani, 2006) and identify perceived peer norms as correlating greatly with alcohol consumption rates (Olds & Thombs, 2001). Overall, the “prevalence of alcohol-based social opportunities on campus contributes to the potency of peer influence on individual attitudes and behaviors” (Borsari & Carey, 2001, p. 392). Both the direct (e.g., “The next time I drink alcohol I would not be able to drink responsibly if an attractive person wanted to buy me a drink”) and indirect (e.g., “The next time I drink alcohol I would not be able to drink responsibly if everyone else was getting drunk”) pressures articulated by participants coincide with research findings noting the significant role of alcohol on the college campus and overall college culture due to the presence of alcoholic beverages at most social gatherings and functions during peer interactions (Thombs, 1999).

A final subtheme emerging from the qualitative phase worth noting is that of a designated caretaker, or “mother hen” to look after inebriated peers. While previous research has documented how college students care for their drunk peers [e.g., “carried home, given a garbage can to throw up in, or extracted from a sexually threatening situation” (Lederman & Stewart, 2005, p. 16)], to date (and to the best of our knowledge), none of the scientific literature associated with collegiate alcohol use/abuse addresses the notion of entrusting one's *decision-making* to another person while under the influence of alcohol. Consequently, further research into the concept and practice of a designated caretaker, and how students identify that person, may prove beneficial in understanding the alcohol consumption-related practices of college students.

While previous investigations have documented that males believe responsible drinking behaviors must occur with significantly less frequency when compared to their female counterparts (Barry & Goodson, 2011a), the quantitative findings from this investigation revealed that they also have less responsible drinking motives and more responsible drinking barriers. These findings coincide with the fact that male college students regularly exceed their female counterparts with regard to the frequency and quantity of alcohol consumption, occurrence of excessive alcohol use, and experienced alcohol consumption-related consequences (Wechsler et al., 2002). As with the documented gender differences, directional effects from this investigation, which classify binge drinkers as having less facilitating factors and more obstacles to drinking responsibly, parallel previous investigations examining one's alcohol consumption in a social setting. Specifically, our findings mirror those of other studies where individuals who consumed a higher number of drinks the last time they were in a social setting (e.g., party, dinner, etc.) had less motivators for, and perceived significantly more barriers to, responsible drinking (Barry & Goodson, 2011b).

## LIMITATIONS

There are important limitations that must be considered in unison with the contributions of this investigation. Specific to the qualitative phase, the lack of gender/ethnic diversity among participants and the small sample size are of particular concern. While transcripts indicate saturation was reached [later focus group sessions support early concepts/ideas and do not provide unique contributions (Lincoln & Guba, 1985)], a more diverse sample could have provided a broader range of insights and perspectives. Furthermore, lack of gender and ethnic diversity could have biased our findings in favor of the perspectives of the majority of participants—Caucasian women.

The most prominent limitation from the quantitative phase was the low survey response rate. Such a low response rate raises questions of the sample's representation. While the demographic distribution of the sample parallels the population from which it was drawn (with regard to both gender and ethnicity), this does not ensure the absence of selection biases. Nevertheless, low response rates with online, electronic surveys of young adults and professionals, has not been uncommon (Chen & Goodson, 2010).

## CONCLUSION

Overall, this research builds upon and enhances previous investigations outlining the behavioral beliefs college students have about responsible drinking (Barry & Goodson, 2011a). To date, this is the first empirical study to determine the various contextual factors that serve to facilitate or impede the responsible drinking practices of college students. Because these factors were examined within a mixed methods framework, findings allow researchers and practitioners to have a more complete understanding of the context in which responsible drinking is practiced and how these practices are influenced. Consequently, this investigation expands and strengthens the limited evidence base associated with responsible drinking. It is important to note, however, that participants did not personally provide insight into what they considered to be responsible drinking. Instead, findings from the qualitative phase of this investigation guided the conceptualization and scope of our responsible drinking construct. Even though systematic steps were taken to develop this construct, it is completely possible that respondents for the quantitative phase had not accounted for interpretations of responsible drinking. Therefore, we are unable to determine how individual differences in terms of defning responsible drinking impacted views of various motivators and inhibitors toward practicing responsible drinking.

Collegiate binge drinking rates are practically the only substance use/misuse rate among young adults that has remained stagnant for nearly the entire past two decades (Schulenderg & Maggs, 2001). While not to dismiss the likelihood that current programs and policies will eventually lead to reductions in these rates, this underscores the importance of developing and instituting harm reduction approaches aimed at minimizing the negative consequences associated with heavy drinking (Marlatt, 1998). Martinic & Leigh, (2004) contend that the fundamental view inherent in an approach to minimizing alcohol consumption-related harm or risk is the assumption that risk is intrinsic to all human activities. Therefore, the objective of this approach in relation to alcohol use is to “ensure that when people drink, they do so in as safe a manner as possible, and that their drinking environ-ment is not conducive to harm” (p. 159). Ensuring that drinking is performed in as safe a manner as possible seems daunting given that getting drunk is an apparent motivator for collegiate alcohol use (Wells, Graham, & Purcell, 2009). Consequently, researchers have called for the development, implementation, and evaluation of effective strategies aimed at minimizing planned intoxication (Wells et al., 2009). We would go one step further and contend further inquiry is necessary before researchers and practitioners will be able to design or implement effective harm reduction strategies aimed at minimizing planned intoxication or teaching responsible drinking.

Considering the scarcity of systematic investigations into responsible drinking practices, or the factors behind one's practice of these behaviors, this initial study opens several avenues for future research. Future inquiries should seek to (a) further flush out how people conceptualize and practice responsible drinking, (b) establish the generalizability of these findings across different academic institutions and geographic regions, and (c) establish additional factors that may serve as a motivator or barrier to responsible drinking. By continuing to examine the contextual factors that influence one's responsible drinking practices, researchers will move one step closer to developing evidence-based strategies designed to promote responsible drinking and diminish the negative health effects associated with excessive alcohol consumption.

## Figures and Tables

**Figure 1:**
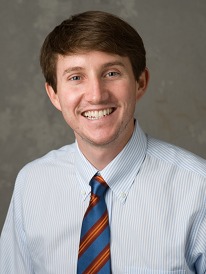


**Figure 2:**
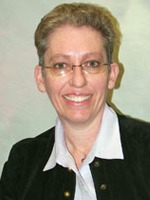


## GLOSSARY

*Responsible drinking*: A construct encompassing many different protecitve behavioral strategies aimed at reducing alcohol-related consequences and harm.

*Partially mixed sequential dominant status design*: A mixed-methods study occuring in two distinct, sequential phases. One of the phases, either qualitative or quantitative, is given greater emphasis.

*Constant comparison model*: A qualitative data analytic technique traditionally outlined in four stages. Overall, these stages encompass comparing and contrasting each fnding or theme as it emerges to the findings/themes that have already been formed. Thus, findings/themes are constantly compared with one another.

## THE AUTHORS

Adam E. Barry, Ph.D., is an Assistant Professor of Health Education and Behavior at the University of Florida. Barry's research focuses broadly upon alcohol use among college students, but extends down two distinct avenues. His first line of inquiry examines how individuals interpret, conceptualize, and practice responsible drinking. His other research investigates the alcohol related issues of military personnel transitioning from service to college. Specifically, Barry's work explores whether student veterans' alcohol-related cognitions and patterns of use differ from those of their nonmilitary peers, as well as whether they also experience a greater proportion of negative outcomes (mental health, social, and academic) as a result of their alcohol use.

Patricia Goodson, Ph.D., is a Presidential Professor for Teaching Excellence at Texas A&M University, in the Department of Health & Kinesiology. Her research interests lie in health promotion theories, sexual health of adults/and adolescents, and public health genomics.
